# Understanding contributors to quality of care in long-term care and changes under COVID-19

**DOI:** 10.1093/geroni/igaa057.3509

**Published:** 2020-12-16

**Authors:** Sara Luck, Katie Aubrecht

**Affiliations:** St. Francis Xavier University, Antigonish, Nova Scotia, Canada

## Abstract

Nursing home facilities are responsible for providing care for some of the most vulnerable groups in society, including the elderly and those with chronic medical conditions. In times of crisis, such as COVID-19 or other pandemics, the delivery of ‘regular’ care can be significantly impacted. In relation to COVID-19, there is an insufficient supply of personal protective equipment (PPE) to care for residents, as PPE not only protects care staff but also residents. Nursing homes across the United States and Canada have also taken protective measures to maximize the safety of residents by banning visitors, stopping all group activities, and increasing infection control measures. This presentation shares a research protocol and early findings from a study investigating the impact of COVID-19 on quality of care in residential long-term care (LTC) in the Canadian province of New Brunswick. This study used a qualitative description design to explore what contributes to quality of care for residents living in long-term care, and how this could change in times of crisis from the perspective of long-term care staff. Interviews were conducted with a broad range of staff at one LTC home. A semi-structured interview guide and approach to thematic analysis was framed by a social ecological perspective, making it possible to include the individual and proximal social influences as well as community, organizations, and policy influencers. Insights gained will improve the understanding of quality of care, as well as potential barriers and facilitators to care during times of crisis.

